# Research on Rhetorical Devices in German: The Use of Rhetorical Questions in Sales Presentations

**DOI:** 10.1007/s10936-022-09874-8

**Published:** 2022-05-21

**Authors:** Jana Neitsch, Oliver Niebuhr

**Affiliations:** 1 Department of English Linguistics (IfLA), University of Stuttgart, Stuttgart, Germany; 2 Centre for Industrial Electronics, Department of Mechanical and Electrical Engineering, University of Southern Denmark, Odense, Denmark

**Keywords:** Rhetorical questions, Information-seeking questions, Prosody, Oral presentation, German

## Abstract

Previous literature recommends using stylistic (or rhetorical) devices in presentations such as rhetorical questions (RQs: *Does anyone want bad teeth?*) to make them more professional, to appear more charismatic, and to convince an audience. However, in oral presentations, it is not only the *what* that matters in using stylistic devices like RQs, but also the *how*, i.e., the RQs’ prosodic realization. To date, however, virtually no handbook on the way of giving a good presentation scrutinizes this prosodic *how*. Therefore, our investigation focuses on the prosodic realization of German RQs in sales pitches. Specifically, we carry out a perception experiment in which 72 listeners rated both the sales pitch and its speaker based on presentations that contained questions that were lexically biased towards a rhetorical interpretation. They were realized with either the prosody of RQs or information-seeking questions (ISQs: *What time is it?*). An additional baseline condition was constituted by regular declarative statements with the corresponding prosody. More precisely, we investigate whether particular identified prosodic realizations—previously found for German RQs and ISQs—meet the listeners’ expectation in the context of a presentation situation. We found that listeners prefer lexically marked RQs that are produced with a prosody that is characteristic of German ISQs. We therefore suggest that handbooks should provide their readers not only with clear definitions of RQs as a stylistic device in presentations (i.e., the *what*), but also with the respective prosodic realization (i.e., the *how*) to make them a properly implemented stylistic device.

## Introduction: Rhetorical Questions and the *What*

The important function of stylistic (or rhetorical) devices in speeches has already been described and applied by ancient Greek philosophers (e.g., Areni, 2003; Roskos-Ewoldsen, 2003) and is nowadays still addressed in handbooks on how to give good oral presentations (e.g., Antonakis et al., 2012; Morgan, 2005; Niebuhr et al., 2017; Roaldsnes, 2017; Rowland, 2021; Soorjoo, 2012; Sprague et al., 2015; Wang et al., 2020), see also the science-based inventory of Charismatic Leadership Tactics (CLTs) put forward by Antonakis et al. (2012). The reason for the amount of literature addressing this issue is that presentations become more and more important in our working life on the one hand, but at the same time, many people fear speaking in public (cf. Slater et al., 2006). To this end, handbooks on giving good speeches usually aim at supporting speakers and giving advice, because a skilled handling of linguistic means in oral presentations makes the presenter appear more interesting, professional, competent, and persuasive (e.g., Petty et al., 1981), and catches the listeners’ attention (Roaldsnes, 2017).

One out of several stylistic devices that is regularly recurring in the literature on charismatic speech is the *rhetorical question* (henceforth: RQ). Generally, RQs can appear as both *wh*-questions and polar questions (e.g., Karagjosova, 2004) as shown in (1) and (2):

1. *wh*-question: Who doesn’t want to go on holiday?
2. Polar question: *Is the Pope Catholic?* (Sadock, 1974: 138)

According to several authors, the interpretation of the examples (1) and (2) as RQs arises from world knowledge (e.g., Rudanko, 1993; Hudson, 1975; see also Neitsch & Marinis 2020), since the general belief is that most people usually love to go on holiday and, even more obvious, that the Pope actually is the head of the Catholic church. Such cases usually tend to be self-certified (see also Schaffer, 2005). Compared to (1) and (2), the examples given in (3) and (4) are less obvious.

(3) Who likes dogs?
(4) Does anyone like football?

Depending on their context, (3) and (4) can be either signaled and interpreted as RQs – e.g., if the answer to the question is clear to all interlocutors (cf. Caponigro & Sprouse, 2007; Sadock, 1971, 1974) – or as genuine information-seeking questions (henceforth ISQs). Because of their ambiguous character, such examples became part of empirical studies that investigated their prosodic realization (in terms of phonetic as well as phonological characteristics) in contexts triggering a rhetorical and an information-seeking interpretation (a.o., Braun et al., 2019; Neitsch et al., 2020; Wochner et al., 2015).

The examples given in (1)–(4) indicate that there are different types of RQs (see also Neitsch, 2019: ch. 3 for an overview), which calls for a clear definition and systematization of RQs, i.e., the *what*. However, an issue which differs across approaches and is still under discussion is whether or not RQs call for an answer. From a semantic/pragmatic point of view, several authors assume that RQs do not have to be answered (see also Banuazizi & Creswell, 1999; Biezma & Rawlins, 2017; Han, 2002), since the answer is implicitly included in an RQ and obvious to all interlocutors (see example (1)) (e.g., Areni, 2003; Sadock, 1971, 1974; Swasy & Munch, 1985; Zillmann & Cantor, 1973). Caponigro and Sprouse (2007) argue that answers to RQs are optional and can be given by the addressee and the speaker. In contrast, in handbooks on giving presentations, readers can find advice suggesting to wait after having posed an RQ until the audience of a presentation is giving an answer (e.g., Morgan, 2005). This discussion has not come to a solution yet. This issue, although it goes beyond the scope of our paper, is an important constituent of future research in order to provide a concise definition of RQs that contributes to the *what*.

Another aspect of a concise definition is to address what RQs can be used for. For instance, van Eemeren and Garssen (2009: 20f.) define RQs as a presentational device that is used in discussions and speeches to engage the audience, for instance, in the courtroom or in parliamentary speeches (see also Ilie, 1995). This perspective follows the approach to characterize RQs as being primarily used as a debating device in conclusions of speeches and in monologues to engage the audience and to stir particular emotions (e.g., Roskos-Ewoldsen, 2003). Braun et al. (2019), on the other hand, exclude RQs that are realized in monologues from their production experiment. This exclusion relies on the idea that by producing RQs, the speaker wants the listener to publicly state that she or he agrees with the speaker on what the answer to the RQ is (i.e., public commitment, e.g., Biezma & Rawlins, 2017). Furthermore, it has been argued that RQs can hardly be realized out of the blue (a.o. Frank, 1990; Gunlogson, 2001; see also Ilie, 1995; Koshik, 2003; Schaffer, 2005; Meibauer, 1986), since they are strongly context-related. In handbooks on giving presentations, however, RQs have been defined as an effective opener (and closer) (Roaldsnes, 2017: 27). Presenters are encouraged not only to begin their presentation with RQs like the one given in (1), but also to make the listeners active participants in the presentation by inviting them to think about the underlying statement that is conveyed (e.g., *Everybody wants to go on holyday, right?*), in this way evoking agreement (e.g., Ilie, 1995).

There is one thing that previous literature agrees on: the verbal power of RQs. Especially in handbooks on giving presentations, the authors state repeatedly that trained, charismatic speakers should make use of stylistic devices such as RQs (e.g., Antonakis et al., 2012; Niebuhr et al., 2017; Soorjoo, 2012; Wang et al. 2020), since “[r]hetorical questions are the verbal equivalent of throwing a wet sponge at a sleeping student […] and a way to make sure your audience is listening” (Roaldsnes, 2017: 27). Especially from the angle of persuasive communication, it is very common that a speaker deliberately phrases a statement as a question (Blankenship & Craig, 2006), which is why RQs became a popular device in sales presentations and advertising slogans (see e.g., Howard, 1988).

However, what is missing in respective handbooks addressing stylistic devices such as RQs is the *how*. That is, they advise their readers to make use of RQs during their presentations but do not tell them *how* to realize RQs prosodically in an efficient way. Therefore, the present paper focuses on the prosody of RQs in sales presentations. To this end, we will make use of the prosodic realization (i.e., the *how*) that was found to be characteristic for German *wh-*RQs and polar RQs in the production experiments mentioned above (e.g., Braun et al., 2019; Wochner et al., 2015). Regarding *wh*-questions, the specific contour was also identified by listeners as RQ prosody in respective perception experiments (Kharaman et al., 2019; Neitsch et al., 2018). Hence, this paper raises the following research question: How do listeners evaluate presenting speakers when the latter realize lexically marked questions with either an RQ- or an ISQ-specific prosody? And how do these evaluations compare to those that result when either type of question is replaced by a declarative statement with a common (unmarked) prosody?

## Background: Rhetorical Questions and the *How*

Previous production experiments on German investigating ambiguous questions similar to those given in (3) and (4) showed that German RQs and ISQs differ with respect to their prosodic properties (e.g., Braun et al., 2019; Wochner et al., 2015). By prosodic properties or prosody, we mean the complex bundle of stress, loudness, intonation, and voice quality that spans and organizes the coinciding string of sound segments and words (e.g., Arvaniti, 2020). Braun et al. (2019) investigated the prosodic realization of string-identical German RQs and ISQs that were presented in two different types of contexts, one triggering an RQ interpretation and one triggering an ISQ interpretation. The participants were presented with a context followed a target question (either *wh-* or polar as in (3) and (4)), which they had to realize as naturally as possible in its previous context. The participants’ realized questions were recorded. The realized contours of the target questions were analyzed using the GToBI annotation guidelines for German intonation (German Tone and Break Indices; e.g., Grice & Baumann, 2002; Grice et al., 1996, 2005), in which **L** indicates **low** targets (i.e., the voice is low) and **H** indicates **high** targets (i.e., the voice is high). An additional asterisk (*) marks which tone (H or L) perceptually shapes the accented syllable. In their analysis of string-identical RQs and ISQs, Braun et al. (2019) focused (among other analyses) on the questions’ nuclear tunes consisting of the nuclear pitch accent and the final boundary tone. The nuclear pitch accent is the one that is perceived as most prominent and represents the most important accent in a phrase (e.g., Grice & Baumann, 2002; Grice et al., 1996, 2005). This final contour was aligned with the sentence-final object noun in the study of Braun et al. (2019 e.g., *celery* in *Who likes celery?* where * marks the tone that is associated with the stressed syllable in *celery*). As a starting point for investigating RQs in oral presentations, the present analysis makes use of those contours that were most often observed in realizations of German RQs (*wh-*: L*+H L-%, polar: L*+H H-%). Concerning ISQs, we chose a representative contour for both question types (i.e. L* H-^H%; see Braun et al., 2019 and Braun et al., 2020 for spontaneous speech). Table 1 illustrates these contours.

**Table 1:** Nuclear pitch accent and final boundary tone of German RQs and ISQs and the corresponding stylized pitch contours, illustrating the contours’ main concept

	*wh*-questions	polar questions
RQ	L* + H L-%	L*+ H H-%
	
ISQ	L* H-^H%	L* H-^H%
	

For *wh*-RQs, the pitch movement is characterized by a low voice on the accented syllable followed by a high voice (L*+ H) which returns to a low level towards the end of the utterance (L-%), whereas ﻿*wh*- and polar ISQs show an accented low syllable (L*) followed by a continuous high rise of the pitch towards the end, indicated in GToBI by an additional upstep (^) in the notation (H-^H%). Polar RQs are characterized by the same pitch accent as in *wh*-RQs. But, unlike the latter, they end in a final plateau (indicated by H-%), with the voice staying almost at the same level until the end of the utterance.

The phonetic analysis reported in Braun et al. (2019) additionally revealed that German RQs were realized with a breathier voice quality, especially in utterance-initial position (i.e., on the *wh*-word in *wh*-questions or on the verb in polar questions), compared to string-identical ISQs (see also Neitsch et al., 2018). Furthermore, RQs showed significantly longer utterance durations than their string-identical counterparts, independently of question type.

In subsequent perception experiments with German listeners, Neitsch (2019: ch. 7, see also Neitsch et al., 2018) reported that *wh*-questions with a L* + H L-% intonation contour, in combination with sentence-initial breathiness, were primarily identified as RQs (see Table 1). The results of another perception experiment by Neitsch and Marinis (2020) shows that the same prosodic profile is perceived as fitting into contexts that trigger a strong speaker attitude with a clear opinion if the lexical content of the *wh*-questions is marked as being inclined towards an RQ interpretation (e.g., *Who likes athlete’s foot?*).

Based on these findings and based on the assumption that RQs make presentations more effective and successful, it is useful to investigate *how* RQs should be realized in presentations in order to provide the listeners with a strong and professional speech that includes an authentic and convincing realization of RQs. Crucially, we ask whether the particular RQ prosodies for *wh*- and polar questions (depicted in Table 1) are also the preferred ones in a presentation in front of an audience, as compared to an ISQ prosody. In addition to the target questions being presented with either an ISQ or RQ prosody, we also inserted statements as a further condition into the elicited speeches. The research question of the present investigation is: How do listeners rate presenting speakers and the presented products when the speakers use (a) declarative statements with a declining unmarked declarative prosody compared to (b) lexically unambiguous RQs that are realized with either an i) RQ prosody or an ii) ISQ prosody?

## Perception Experiment

To investigate the research question outlined above, a perception experiment was designed and conducted. Participants were, on an auditory basis, presented with two different sales presentations that were previously recorded. Each of the two presentations was recorded by two speakers, one male and one female speaker. Both were phonetically trained. Participants were asked to rate the presenting speakers and the products that occurred in the presentations.

### Materials

As a first step, two presentations were designed, each of them describing a new product: a new, healthier toothpaste and a new kind of drinking chocolate. The choice of the two products was such that each of the participants would be familiar with their basic properties, while understanding at the same time the products innovative features: A toothpaste that does not contain any artificial aromas, preservatives or parabens, but only natural ingredients instead; and a drinking chocolate that contains Guarana instead of caffeine and was manufactured under better humane trade relations than similar products.

The written script to each of the two presentations consisted of four paragraphs that meet the basic requirements of a standard sales presentation (Bird, 2012): a teaser, a short comparison with similar products, the main paragraph that listed the competitive advantages of the product, and a final closing paragraph with an implicit call to action. In each of the presentations, five target questions (3 *wh*- and 2 polar) were inserted, one in the teaser paragraph, one in the comparison paragraph, two in the main paragraph, and one in the closing paragraph. The two authors of this paper (both are native speakers of German) opted for the question type (*wh*- vs. polar) whose syntactic structure and rhythm pattern that matched best with the given context.

In order to get as close as possible to the question strings of previous investigations (a.o. Braun et al. 2019, Neitsch 2019, Wochner et al. 2015), all polar questions were designed such that the verb appeared in the first position (V1) followed by the German modal particle *denn* and the indefinite subject *anyone* (*Will denn jemand (…)* ‘Wants PRT anyone (…)’). *Wh*-questions were designed such that the lexical string consisted of the *wh*-pronoun *wer* ‘who’, followed by the verb. The third constituent of *wh*-questions was the German modal particle (PRT) *denn* (lit. ‘then’, *Wer will denn (…)* ‘Who wants PRT (…)’). If necessary, another constituent such as ‘das’ (lit. ‘this’, ‘that’; e.g., ‘Who wants PRT to miss this?’) was inserted between verb and particle in order to create a suitable syntactic and rhythmic structure.

Additionally, we designed a version of each presentation as reference condition with declarative statements instead of all five questions (i.e., *Zähneputzen ist wichtig; Es will ja keiner schlechte Zähne.* ‘Brushing the teeth is important; Nobody wants bad teeth.’). The design resulted in three presentation conditions: question condition with RQ prosody, question condition with ISQ prosody, and declarative condition with a typical pitch declination (note that the main acoustic correlate of perceived pitch in human speech is the fundamental frequency: F0; Niebuhr et al., 2021; we will use F0 henceforth to refer to pitch). Since the present investigation focuses on the prosody of RQs in sales presentations, the contexts in which they were embedded were designed such that they would lead to unambiguous RQ interpretations (see Table A1 in the Appendix for the presentations) as is shown in the examples (5) and (6) for *wh-* and polar questions, respectively. (7) shows the respective declaratives of (5) and (6).

(5) *wh*-question:

Zähneputzen ist wichtig; *Wer will denn schlechte Zähne?*

‘Brushing the teeth is important; Who wants bad teeth?’

(6) Polar question:

Anders als Koffein ist Guarana jedoch leichter bekömmlich […] und ist auch bei regelmäßigem Genuss absolut unbedenklich für den Organismus. *Möchte denn jemand dem Organismus schaden?*

‘Unlike caffeine, guarana is easier to digest [...] and is absolutely harmless to the organism even with regular consumption. Does anyone want to harm the organism?’

(7) declaratives:

*Es will ja keiner schlechte Zähne.* **‘**Nobody wants bad teeth.’

*Es will ja keiner dem Organismus schaden.* ‘Nobody wants to harm the organism.’

The phonetically trained speakers (female: 30 years; male: 40 years) recorded both sales presentations with questions: once with *wh-* and polar questions and RQ prosody (including initial breathiness) and once with *wh-* and polar questions and ISQ prosody (in modal voice quality).

The recorded presentations were cut with Praat (Boersma & Weenink, 2020) and manipulated such that there were three versions of each of the presentations per speaker – a version with questions and RQ prosody, a version with the same string-identical questions and ISQ prosody, and a version with declaratives and a declining, terminally falling prosody. The manipulation was conducted such that the temporal boundaries of the target-sentence renderings were marked in the signal and then, for each version, the respective target sentences (with RQ prosody, with ISQ prosody, declaratives) were cut into the sales presentation framework. Where required, F0 resynthesis or intensity normalization procedures were applied to smooth the prosodic patterns at both edges of the inserted target sentences.

**Fig. 1:**
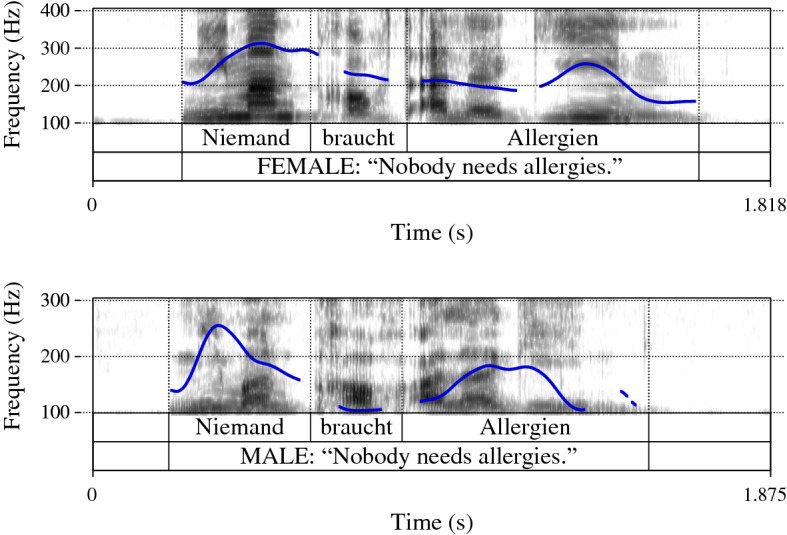
Declaratives realized by female speaker (upper panel) and male speaker (lower panel)

Additionally, both speakers recorded a version of each presentation with declaratives instead of questions (i.e., Zähneputzen ist wichtig; *Es will ja keiner schlechte Zähne*. ‘Brushing the teeth is important; *Nobody wants bad teeth.*’; see Fig. 1 and example (7)). All recorded declaratives showed a prosody similar to those illustrated in Fig. 1, indicating the characteristic F0 declination (F0 peaks become lower and lower towards the end of an utterance, see e.g., Oppenrieder, 1989: 245ff. for an overview and references therein).

Overall, the questions that were recorded in the two different prosodic ways reflected the differences that were previously identified to characterize RQs as opposed to string-identical ISQs in German (e.g., Braun et al., 2019; Neitsch et al., 2018). More specifically, in initial position, voice quality measurements using Harmonics-to-Noise Ratio (HNR in dB) indicated that both speakers realized RQs with a breathier voice quality than ISQs (note that the lower the HNR value, the higher the breathiness and vice versa). The HNR averages of the female speaker were 10.64 dB for RQs and 17.67 dB for ISQs and those of the male speaker were 11.77 dB for RQs and 15.02 dB for ISQs (*p*  < 0.05 for both speakers). In addition, the recorded RQs showed longer sentence durations than ISQs (female speaker’s average: 1.95 s for RQs and 1.66 s for ISQs; male speaker’s average: 1.80 s for RQs and 1.51 s for ISQs; *p*  < 0.05 for both speakers). The final object noun showed the typical extra lengthening in the RQ realization (female speaker’s average for RQs: 0.95 s vs. ISQs: 0.74 s; male speaker’s average for RQs: 0.83 s vs. ISQs: 0.66 s; *p* =  < 0.05 for both speakers).

### Procedure

Participants took part in a web-based online survey. Each of them was randomly assigned to one of 12 experimental lists (2 speakers × 2 presentations × 3 prosodies) containing one presentation in one prosody version of the male speaker and the other presentation of the same prosody version of the female speaker (e.g., drinking chocolate of male speaker with RQ prosody, toothpaste of female speaker with RQ prosody). Prior to the experiment, participants were asked to answer questions concerning their age, education, native language as well as other languages that they had learned before the age of six. Moreover, participants were asked to check the functionality and volume of their loudspeakers. They were asked to set the volume to a comfortable loudness.

In the actual experiment, participants used a web player with a play button embedded in the survey. Participants were instructed to listen once to the recorded presentation by clicking on the play button with their computer mouse. While they listened to the presentation, they were presented with a picture that was related to the product. On the next page of the survey, participants were asked to judge the speaker’s performance and perceived traits on 6-point scales ranging from ‘not at all’ (1) to ‘very strong’ (6). Eleven attributes were used, taking into account the MASCharP Scale for rating perceived charisma by D’Errico et al. (2013): *sympathy, conviction, enthusiasm, determination*, *visionary*, *inspiration*, *attractiveness, confidence, professionalism, charisma*, and *credibility*. During this rating process, they were allowed to listen to the presentation again. Only after a 1-to-6 rating on each of the scales had been made, participants were allowed to proceed. In a second rating block, participants were asked to answer two further questions on the same sales pitch using a slider scale (1 to 10 with 10 indicating the highest probability): ‘How probable is it that you would invest in this product?’ and ‘How probable is it that this product is successful?’. Only if participants moved the slider away from its middle position in the direction of either ‘not at all’ or ‘highly probable’, they were able to proceed with the survey.

### Participants

Seventy-two monolingual native speakers of German (40 female, 32 male, average age = 33.27, SD = 11.40) participated in the experiment (6 participants × 12 experimental lists). All of them were unaware of the purpose of the study, and none of them reported any speaking or hearing disorders. Overall, the participants showed a large variation regarding their education and profession.

### Data Analysis

Participants’ ratings were split into two blocks (block A: rating on 6-point scale; block B: success of product) and statistically analyzed using R (R Core Team 2013). Mixed effects regression models were calculated by including the rating values for each of the eleven attributes (i.e., *sympathy, conviction, enthusiasm, determination*, *visionary*, *inspiration*, *attractiveness, confidence, professionalism, charisma*, and *credibility*) as dependent variables, and *speaker* (female vs. male) and *condition* (ISQ, RQ, declarative) as independent variables.

Since both the ISQ and the RQ condition were based on exactly the same string-identical target questions, they were equally distributed across both presentation conditions, while presentations based on declaratives constituted a third condition. As a first step and in order to focus on the overall rating differences that emerged between RQ and ISQ prosodies, we will not further distinguish between the underlying question type, i.e., *wh-* and polar question.

## Results

### Attribute Ratings

**Fig. 2:**
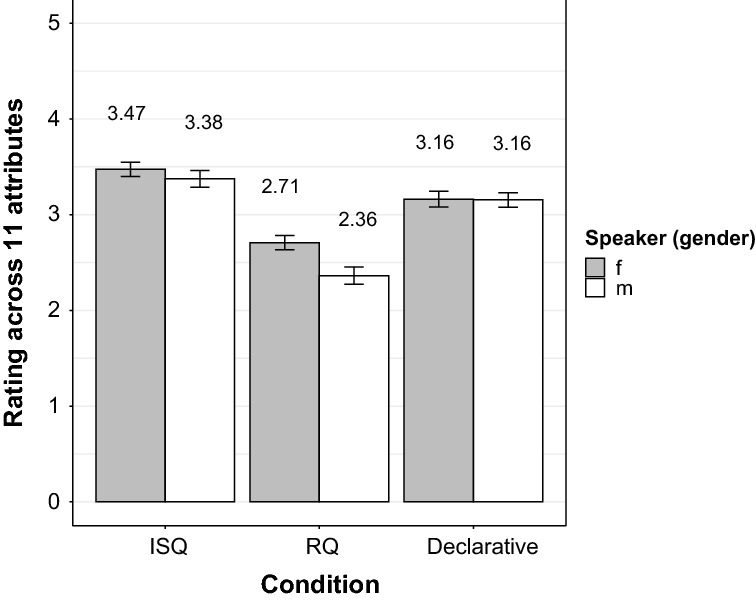
Mean rating across all 11 attributes (y-axis) in the three experimental conditions (x-axis: ISQ prosody vs. RQ prosody vs. declaratives) per speaker gender. Ratings are shown here from 0 to 5 (y-axis) to enhance the differences

Participants’ ratings of the speaker attributes along the 6-point scales showed an interaction between speaker and condition (ß = 0.34, *SE* = 0.16, *t* = 2.08, *p* = 0.04, see Fig. 2). It resulted from an advantage (more positive attribute ratings) of the female speaker over the male speaker for all eleven attributes. Therefore, the data were split up according to (﻿a) *condition* and (b) *speaker*. The analysis of the subset (a) showed an advantage of the female speaker with respect to the RQ-prosody condition, which obtained overall higher ratings across all eleven attributes compared to that of the male speaker (ß = 0.34, *SE* = 0.12, *t* = 2.96, *p* = 0.003). In contrast, the effect of *speaker* wa﻿s absent for the ISQ-prosody condition as well as for declaratives (both *p*-values > 0.38).

Regarding the subset concerning (b), the results of the female speaker indicated that – compared to the RQ condition – ratings for both the ISQ-prosody condition (ß = 0.77, *SE* = 0.11, *t* = −7.07, *p* < 0.0001) and the declarative condition (ß = 0.45, *SE* = 0.11, *t* = 4.20, *p* < 0.0001) were significantly higher across all eleven attributes. The ISQ-prosody condition also obtained significantly higher ratings than the declarative condition across all attributes (ß = 0.31, *SE* = 0.11, *t* = 2.87, *p*  = 0.004).

Results for the male speaker indicated that – compared to the RQ condition – ratings for both the ISQ-prosody condition (ß = 1.01, *SE* = 0.12, *t* = 8.44, *p* < 0.0001) and the declarative condition (ß = 0.79, *SE* = 0.12, *t* = 6.61, *p* < 0.0001) were significantly higher across all eleven attributes. However, the difference between the ISQ condition and the declarative condition only approached significance (*p* = 0.07), indicating higher ratings for the ISQ condition than for the declarative condition.

**Fig. 3:**
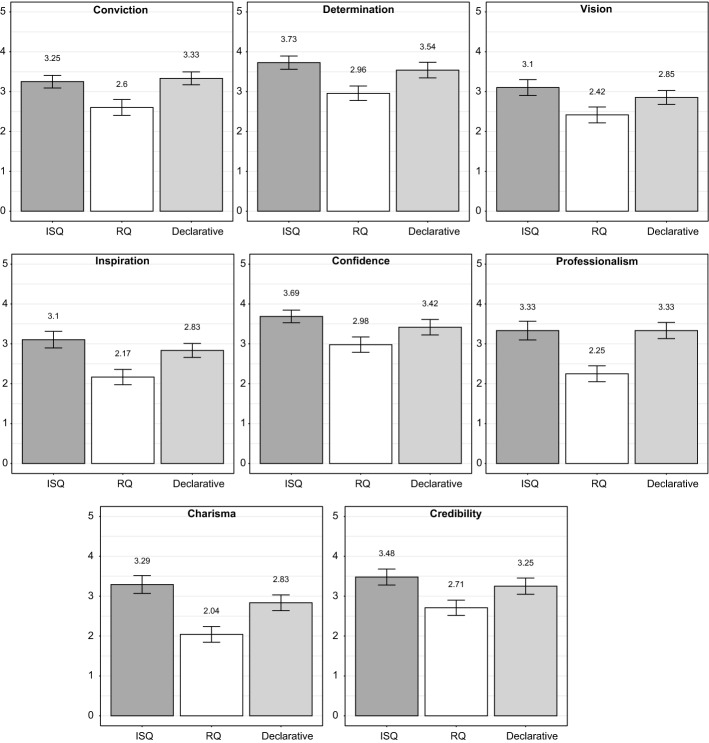
Mean ratings for single attributes (y-axis) across all three presentation conditions (x-axis). Ratings are shown here from 0 to 5 (y-axis) to enhance the difference

To understand the overall picture of these findings, we take a look at the participants’ ratings for each attribute separately (see Fig. 3 for all results). With respect to *conviction*, the speakers were rated as significantly more convincing if they used the ISQ prosody (ß = 0.65, *SE* = 0.25, *t* = 2.64, *p* = 0.009) or declaratives (ß = 0.73, *SE* = 0.25, *t* = 2.98, *p* = 0.003) in their sales pitches, compared to the RQ prosody. There was no significant difference between pitches with ISQ prosody and declaratives (*p* = 0.73).

Regarding *determination*, the speakers were rated as being more determined if they realized both ISQs (ß = 0.77, *SE* = 0.26, *t* = 3.01, *p* = 0.003) and declaratives (ß = 0.58, *SE* = 0.26, *t* = 2.28, *p* = 0.02) in their presentations, compared to pitches including RQs. Again, there was no significant difference between the ISQ condition and the declarative condition (*p* = 0.47).

The two speakers were rated as more *visionary* if they realized the target questions with ISQ prosody (ß = 0.69, *SE* = 0.27, *t* = 2.56, *p* = 0.01) rather than RQ prosody in their presentation. There was no difference between the declarative condition and the RQ condition (*p* = 0.11) or between the declarative condition and the ISQ condition (*p* = 0.36).

For *inspiration*, we found that speakers were rated as more inspiring if their presentations contained questions with ISQ prosody (ß = 0.94, *SE* = 0.27, *t* = 3.48, *p* = 0.0007) or declaratives (ß = 0.67, *SE* = 0.27, *t* = 2.47, *p* = 0.01), compared to when they contained questions that were realized with the RQ prosody. There was no significant difference between the ISQ condition and the declarative condition (*p* = 0.32).

Results concerning *confidence* showed that the speakers were rated as being more confident if they realized questions with the ISQ prosody compared to the RQ prosody (ß = 0.71, *SE* = 0.26, *t* = 2.76, *p* = 0.007). The declarative condition realized in the presentations by the speakers did not differ significantly from both the ISQ condition and the RQ condition (both *p*-values > 0.10).

Results for *professionalism* showed that the speakers were generally rated as more professional if they realized their target questions with ISQ prosody (ß = 1.08, *SE* = 0.30, *t* = 3.62, *p* < 0.0005) or used declaratives (ß = 1.08, *SE* = 0.30, *t* = 3.62, *p* < 0.0005), compared to target questions with RQ prosody. There was no difference between the ISQ condition and the declarative condition (*p* = 1.0, in fact both values were identical).

Results for *charisma* indicated that speakers were perceived as more charismatic if they relied on either the ISQ condition (ß = 1.25, *SE* = 0.29, *t* = 4.32, *p* < 0.0001) or the declarative condition (ß = 0.79, *SE* = 0.29, *t* = 2.73, *p* = 0.007), compared to the RQ condition. There was no difference between the ISQ condition and the declarative condition (*p-*value = 0.12).

With respect to the *credibility* of the speakers, results showed higher ratings if the presentations contained either question realizations with ISQ prosody (ß = 0.77, *SE* = 0.28, *t* = 2.76, *p* = 0.007) or declaratives (ß = 0.54, *SE* = 0.28, *t* = 1.94, *p* = 0.05), both compared to the RQ condition. There was no additional difference between the ISQ condition and the declarative condition (*p-*value = 0.41).

**Fig. 4:**
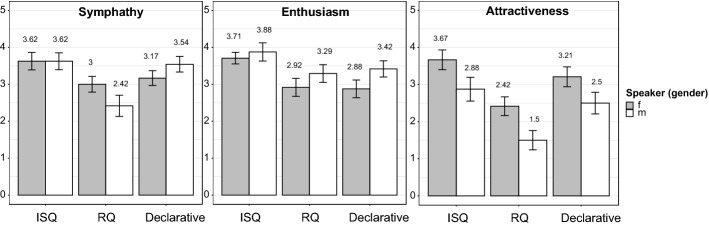
Mean ratings for single attributes (y-axis) across all three presentation conditions (x-axis) for both speakers. Ratings are shown here from 0 to 5 (y-axis) to enhance the difference

There were findings for which the factor *speaker* (male vs. female) caused an additional statistical difference in the ratings (see Fig. 4).

For instance, regarding *sympathy*, there was an interaction between *condition* and *speaker* (ß = 0.96, *SE* = 0.45, *t* = 2.11, *p* = 0.04). Splitting up the data according to *speaker*, results indicated that the male speaker was rated as significantly more sympathetic if he realized his target questions with an ISQ prosody (ß = 1.13, *SE* = 0.34, *t* = 3.31, *p* = 0.001) or used declaratives (ß = 1.21, *SE* = 0.34, *t* = 3.56, *p* = 0.0007), both compared to target questions with RQ prosody. There was no additional difference between the ISQ condition and the declarative condition (*p* = 0.81). Regarding the female speaker, ratings on sympathy were higher if she realized target questions with ISQ prosody compared to RQ prosody (ß = 0.63, *SE* = 0.30, *t* = 2.08, *p* = 0.04), but there was no additional difference between the ISQ condition and the declarative condition (*p* = 0.13), or between the declarative condition and the RQ condition (*p* = 0.58).

Results obtained for *enthusiasm* showed no interaction between *speaker* and *condition* (*p* > 0.40), but an effect of *condition*, indicating that presentations were rated as significantly more enthusiastic if the speakers produced target questions with an ISQ prosody rather than an RQ prosody (ß = 0.69, *SE* = 0.22, *t* = 3.10, *p* = 0.002). Additionally, the ISQ condition showed significantly higher ratings than the declarative condition (ß = 0.65, *SE* = 0.22, *t* = 2.91, *p* = 0.004), but ratings related to presentations with declaratives did not differ from presentations containing target questions with an RQ prosody (*p* = 0.85). The additional effect of *speaker* showed higher *enthusiasm* ﻿ratings for the male than for the female speaker (ß = 0.36, *SE* = 0.18, *t* = 1.99, *p* = 0.05).

Results obtained for *attractiveness* showed no interaction between *speaker* and *condition* (*p* > 0.70), but an effect of *condition*, showing that speakers were rated as more attractive if they produced their target questions with ISQ prosody (ß = 1.31, *SE* = 0.27, *t* = 4.85, *p* < 0.0001) or if they used declaratives (ß = 0.90, *SE* = 0.27, *t* = 3.31, *p* = 0.001) as compared to target questions with RQ prosody. There was no difference between the ISQ condition and the declarative condition (*p* = 0.13). An effect of *speaker* indicated a higher level of attractiveness for the female than for the male speaker (ß = 0.81, *SE* = 0.22, *t* = 3.65, *p* < 0.0005). The factor *product* (chocolate vs. toothpaste) showed no effect on the results (all *p-*values > 0.8) for any of the previous models.

### Probability Ratings

As described above, the second part of participants’ task was to assess (i) if they would invest in the product (henceforth: *probability of product investment*), and (ii) if both the speaker and the product would become successful (henceforth: *probability of success*). To this end, in order to achieve a better specification, *product* was included as a further variable in the statistical model.

**Fig. 5:**
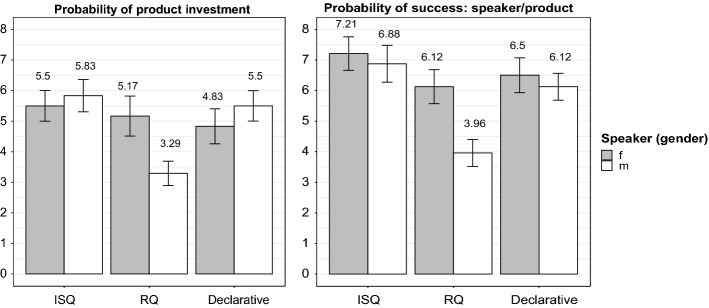
Average probability ratings (y-axis) across all three presentation conditions (x-axis) for both speakers. Ratings are shown here from 0 to 8 (y-axis) to enhance the difference

Regarding (i), participants’ ratings showed an interaction between *condition* and *speaker* (ß = −2.54, *SE* = 1.04, *t* = −2.44, *p* = 0.02, see Fig. 5). Splitting up the dataset according to *speaker*, we find higher ratings for the male speaker if he realized his target questions with the ISQ prosody (ß = 2.54, *SE* = 0.67, *t* = 3.82, *p* = 0.0003) or used declaratives (ß = 2.21, *SE* = 0.67, *t* = 3.32, *p* = 0.001), both compared to the RQ-prosody condition. However, there was no additional difference between the ISQ condition and the declarative condition (*p* = 0.62). Regarding the dataset of the female speaker, there was no difference at all (all *p*-values > 0.41).

Splitting up the dataset according to *condition*, the results for the female speaker regarding presentations containing target questions with RQ prosody showed significantly higher ratings than for the male speaker (ß = 1.88, *SE* = 0.75, *t* = 2.51, *p* = 0.02), an effect which was absent for the ISQ and declarative conditions (both *p*-values = 0.38). Again, there was no effect of *product* in either of the models (all *p-*values > 0.14).

With respect to (ii), participants’ ratings showed a nearly significant interaction between *condition* and *speaker* (*p* = 0.08). Therefore, the model was reduced to the possible main effects. Results indicate that both the *product* as well as the *speaker* were assumed to become more successful if participants listened to the female speaker compared to the male speaker (ß = 0.96, *SE* = 0.43, *t* = 2.24, *p* = 0.03; Fig. 5). Additionally, both the *speaker* and the *product * were assumed to become more successful if presentations were realized with questions and an ISQ prosody (ß = 2.00, *SE* = 0.52, *t* = 3.82, *p* = 0.0002) or declaratives (ß = 1.27, *SE* = 0.52, *t* = 2.43, *p* = 0.02) as compared to questions with RQ prosody. There was no difference between the ISQ condition and the declarative condition (*p* = 0.17) and no difference in the models regarding the *product* that was advertised in the presentation (all *p*-values > 0.14).

## Discussion

The present results showed that the different products that were presented did not have any effect on the results, thus underlining the goodness of the designed experimental material. The overall result for participants’ ratings of the attributes concerning the two presenting speakers indicated that the female speaker had an advantage over the male speaker, particularly with respect to the RQ condition. That is, the female speaker achieved higher ratings than the male speaker across all eleven attributes when producing target questions with an RQ prosody. Within each of the speaker-related subsets, the results of the female speaker indicated that – across all attributes – ratings were significantly higher for presentations that relied on declaratives or questions with an ISQ prosody than for presentations that contained target questions in the prosodic RQ shape, with an additional advantage of the ISQs over their declarative counterparts. The same result emerged for the male speaker, apart from the fact that the higher ratings for presentations using target questions with ISQ prosody compared to presentations including declaratives only approached significance.

Regarding the individual attributes that were rated in the experiment, results indicated no effect of speaker for eight out of eleven attributes (i.e., *conviction*, *determination*, *inspiration*, *visionary*, *confident*, *professionalism*, *charisma*, *credibility*). For all of them, we found that both speakers achieved higher ratings if they realized their target questions with an ISQ prosody than with an RQ prosody, and that presentations containing target questions with an ISQ prosody never differed significantly from the declarative condition. However, the declarative condition showed an additional difference to the RQ-prosody condition for *conviction*, *determination*, *inspiration*, *professionalism*, *charisma*, and *credibility*. That is, for all of these six attributes, both the ISQ and declarative condition were equivalent with respect to how the speaker was perceived in her/his presentation. The absence of a possible threefold gradation in these six cases indicates that a “genuine question” in terms of a question with ISQ prosody, is about equally as acceptable as a declarative in a sales-pitch setting. A further important point is that speakers using declaratives or target questions with an RQ prosody in their sales pitches sounded comparable in terms of *vision* and *confidence*. In other words, to make sure to be perceived as a visionary and confident speaker, declaratives should be avoided (or interspersed with any type of question), since their ratings fall in-between the ratings for presentations containing target questions with ISQ prosody and RQ prosody.

There were also findings for which speaker (male vs. female) made an additional difference with respect to the ratings for *sympathy*, *enthusiasm*, and *attractiveness*. For all three attributes our findings showed higher ratings for speakers realizing an ISQ prosody compared to an RQ prosody. Regarding *sympathy*, the male speaker appeared to be more or about equally sympathetic if he realized his target questions with an ISQ prosody or a declarative prosody, respectively. In contrast, the only difference for the female speaker was the rating regarding *sympathy* between ISQs and RQs. Moreover, we found higher *enthusiasm* ratings for the male than for the female speaker, but higher ratings for *attractiveness* for the female than for the male speaker.

With respect to the *probability of product investment* and the *probability of success*, the findings showed that the male and female presenter were perceived differently depending on the respective experimental condition (i.e., ISQ prosody, declarative, RQ prosody). For instance, even for the RQ condition that was generally rated lowest, ratings concerning the female speaker showed an advantage over those of the male speaker. Also, regarding the probability of success, both the product and the speaker were assessed as being more successful, if participants listened to the female speaker. Nevertheless, both speakers were always on the “safe side” (in terms of ratings in the top half of the scale) if they realized the target questions in their presentations with ISQ prosody.

In summary, our findings suggest that to be perceived as a good speaker in oral presentations, speakers should realize questions that function as RQs with an ISQ prosody. Using the RQ prosody in presentations like sales pitches did not only cause the lowest ratings across all tested attributes. It also led to the lowest evaluation with respect to the probability of product investment and success of both the speaker and the product. This suggests that questions that are meant to be interpreted as RQs are not automatically acceptable in all settings, such as a presentation situation, if they show the prosodic realization that was previously found to be common for German RQs. Our findings hence indicate that the prosody of RQs seems to be situation-dependent and that a definition of RQs based on their prosody cannot hold for all situations. Instead, researchers working on the investigation of prosody need to focus on the prosody of RQs and the function of prosody in different situations.

## Conclusion

Overall, based on our findings, we suggest that handbooks on rhetoric should not simply advise their readers to use RQs in presentations without providing them with additional information about *what* RQs are and *how* they are to be realized prosodically. Based on the present findings suggesting the use of an ISQ prosody (i.e., modal voice quality with nuclear L* H-^H%) for lexically unambiguous RQs in German, the authors of handbooks should define RQs on the basis of their prosody. Moreover, the results suggest that target questions with an RQ interpretation in presentations are used and realized differently than RQs in other settings. In sales presentations, RQs can be used as openers even though RQs have previously been defined as questions that cannot be realized out of the blue (e.g., Gunlogson, 2001: 2).

Our results further suggest that using declaratives can, in many cases, be the better option than using questions (with RQ prosody) in sales presentations. However, future studies will also have to investigate the precise prosodic realization of declarative statements as a stylistic tool in presentation situations. Additionally, future investigations should take a closer look at the differences between *wh-* and polar questions in presentation situations or different communication settings in general.

Generally, we assume that depending on the setting (e.g., close friends vs. foreign audience, colloquial vs. professional) in which RQs are used, prosodic variation make RQs a highly complex, under-researched phenomenon. Prosody not only signals the communicative function of an utterance (inviting, commanding, seeking information), but also the respective affective state of the speaker. If the realized prosody (of RQs) contrasts with the expected aim of a polite invitation of the audience to like or buy a particular product (which is usually the aim of sales pitches), the speakers receive low ratings for their performance and for the product/idea that they pitch. This suggests that results concerning prosody investigated in lab situations cannot necessarily be generalized to other non-lab situations, which, in turn, stresses the need for (a) investigations in various contexts and (b) for experiments that address the interplay between context, utterance content, and prosody in more detail.

Finally, since RQs are not the only stylistic device that make a presentation more professional (cf. the CLT list of Antonakis et al., 2012), the prosodic realization of other devices (e.g., alliterations, contrasts, enumerations) need to be analyzed in future investigations as well.

## Appendix

See Table A1.

**Table A1:** The recorded presentations with target questions or declaratives

**Der natürliche Wachmacher**
Jeder liebt Schokolade; **Wer will das denn abstreiten?/Niemand will das abstreiten.** Noch dazu, wenn sie wach macht und die Sinne weckt. Unsere Trinkschokolade, aus edlem lateinamerikanischen Kakao, enthält belebendes Guarana. Guarana ist eine koffeinhaltige Pflanze aus Brasilien. Ihr Wirkstoff erfrischt und macht auf natürliche Weise wach. Anders als Koffein ist Guarana jedoch leichter bekömmlich, wirkt 4 × länger als Koffein und ist auch bei regelmäßigem Genuss absolut unbedenklich für den Organismus. **Möchte denn jemand dem Organismus schaden?/Es will ja keiner dem Organismus schaden.** Mit unseren beiden Sorten sprechen wir Kinder und Genießer an, die auch am Späten Abend ihre Tasse Kakao trinken wollen. **Wer will denn darauf verzichten?/Niemand will darauf verzichten.** Unsere Trinkschokolade ist für alle Menschen, die Schokolade lieben und für den natürlichen Frischekick zwischendurch. Unser Produkt richtet sich auch an alle Menschen die Kaffee nicht mögen oder vertragen und bietet die ideale Alternative. Zudem legen wir Wert auf ökologischen Anbau sowie auf eine transparente und faire Handelsbeziehung. **Will denn jemand menschenunwürdige Handelsbeziehungen?/Es will ja keiner menschenunwürdige Handelsbeziehungen.** Darin sehen wir unseren persönlichen Beitrag zum Schutz der Umwelt und zur Realisierung besserer Arbeits- und Lebensstandards in den Anbauländern. Mit dem Kauf unseres Produktes unterstützen Sie ein kompetentes und dynamisches Team, das weltweit führend in der Entwicklung von Guarana-Trinkschokolade ist. Probieren Sie also jetzt unsere einzigartige Trinkschokolade. **Wer möchte das denn verpassen?/Niemand will das verpassen** **The natural stimulant** “Everyone loves chocolate; **who wants to deny that?/Nobody wants to deny that**. Especially when it wakes you up and awakens your senses. Our drinking chocolate, made from fine Latin American cocoa, contains invigorating guarana. Guarana is a caffeinated plant from Brazil. Its active ingredient refreshes and awakens in a natural way. Unlike caffeine, guarana is easier to digest, works 4 × longer than caffeine and is absolutely harmless to the organism even with regular consumption. **Does anyone want to harm the organism?/Nobody wants to harm the organism.** With our two varieties we address children and connoisseurs who also want to drink their cup of cocoa late in the evening. **Who wants to do without it?/Nobody wants to do without it.** Our drinking chocolate is for everyone who loves chocolate and for a natural freshness kick in between. Our product is also aimed at everyone who does not like or handle coffee and offers the ideal alternative. We also attach great importance to ecological cultivation and a transparent and fair-trade relationship. **Does anyone want inhumane trade relationships?/Nobody wants inhumane trade relationships.** This is where we see our personal contribution to protecting the environment and realizing better working and living standards in the producing countries. With the purchase of our product you support a competent and dynamic team that is the world leader in the development of guarana drinking chocolate. So try our unique drinking chocolate now. **Who wants to miss that?/Nobody wants to miss it.”**
**Die alternative Zahncreme**
Zähneputzen ist wichtig; **Will denn jemand schlechte Zähne?/Es will ja keiner schlechte Zähne.** Zahn-cremes müssen ordentlich säubern und vor Karies schützen. Dafür sorgt der Inhaltsstoff Fluorid. Doch aus-gerechnet diese Substanz kann auch den Zahnschmelz angreifen. Zudem geraten immer mehr Zahncremes wegen ihrer synthetischen Konservierungsstoffe, sogenannter Parabene, in Verruf. Sie stehen im Verdacht Allergien beim Verbraucher auszulösen. **Wer braucht denn Allergien?/Niemand braucht Allergien.** Unser Team besteht aus hochqualifizierten Zahnärzten, Ernährungs- und Lebensmittelwissenschaftlern, und wir entwickeln unser Produkt nach dem neuesten Stand der Forschung und Technik. Wir bieten Ihnen eine natürliche Alternative zu herkömmlichen Zahncremes ohne Zusatz von Fluorid und künstlichen Konservierungsstoffen. Unsere patentierte Rezeptur enthält lediglich acht naturreine Zutaten, die aus nachhaltigem und biodynamischem Anbau stammen. Wir verwenden hochwertigen Grapefruitkernextrakt und Vitamin E als Konservierungsstoffe und wir setzen auf naturreinen Birkenzucker als natürlichen Abriebersatz. **Wer braucht denn da noch Parabene?**/**Niemand braucht da noch Parabene.** Langzeittests haben bereits gezeigt, dass nicht nur das Putzergebnis überzeugt, sondern auch die Risiken für Karies, Zahnfleischprobleme und Zahnstein innerhalb von zwei Wochen um 80% reduziert werden. Auch beim Geschmack müssen Sie auf Grund natürlicher Aromastoffe keinerlei Abstriche machen. **Will denn jemand künstliches Aroma?/Es will ja keiner künstliches Aroma.** Unser Produkt ist für alle, die nach einer natürlichen Alternative für handelsübliche Zahncremes suchen und dabei auf den erfrischenden Geschmack von Salbei und Minze nicht verzichten wollen. **Wer möchte das denn versäumen?/Niemand will das versäumen** **The alternative toothpaste** “Brushing your teeth is important; **does anyone want bad teeth?/Nobody wants bad teeth.** Toothpaste must clean properly and protect against caries. This is ensured by the ingredient fluoride. But this substance of all things can also attack tooth enamel. In addition, more and more toothpastes are being discredited because of their synthetic preservatives, so-called parabens. They are suspected to trigger allergies in the consumer. **Who needs allergies?/Nobody needs allergies**. Our team consists of highly qualified dentists, nutritionists and food scientists, and we develop our product according to the latest research and technology. We offer you a natural alternative to conventional toothpastes without the addition of fluoride and artificial preservatives. Our patented recipe contains only eight naturally pure ingredients that come from sustainable and biodynamic cultivation. We use high-quality grapefruit seed extract and vitamin E as preservatives, and we rely on pure birch sugar as a natural substitute for abrasion. **Who needs parabens anymore?/Nobody needs parabens anymore.** Long-term tests have already shown that not only the cleaning result is convincing, but also the risks for caries, gum problems and tartar are reduced by 80% within two weeks. You do not have to compromise on taste due to natural flavors. **Does anyone want artificial aroma?/Nobody wants artificial aroma.** Our product is for everyone who is looking for a natural alternative to commercially available toothpastes and does not want to do without the refreshing taste of sage and mint. **Who wants to miss that?/Nobody wants to miss it.**”

## Declarations

### Data Availability

The datasets used and analyzed during the study are available from the corresponding author on reasonable request, since we are not allowed to save and store our data online.

### Informer content

Informed consent was obtained from all individual participants of the study.
